# The impact of life course exposures to neighbourhood deprivation on health and well-being: a review of the long-term neighbourhood effects literature

**DOI:** 10.1093/eurpub/ckz153

**Published:** 2019-10-02

**Authors:** Stephen Jivraj, Emily T Murray, Paul Norman, Owen Nicholas

**Affiliations:** 1 UCL Institute of Epidemiology and Health Care, London, UK; 2 School of Geography, University of Leeds, Leeds, UK; 3 UCL Department of Statistical Science, London, UK

## Abstract

**Background:**

In this review article, we detail a small but growing literature in the field of health geography that uses longitudinal data to determine a life course component to the neighbourhood effects thesis. For too long, there has been reliance on cross-sectional data to test the hypothesis that where you live has an effect on your health and well-being over and above your individual circumstances.

**Methods:**

We identified 53 articles that demonstrate how neighbourhood deprivation measured at least 15 years prior affects health and well-being later in life using the databases Scopus and Web of Science.

**Results:**

We find a bias towards US studies, the most common being the Panel Study of Income Dynamics. Definition of neighbourhood and operationalization of neighbourhood deprivation across most of the included articles relied on data availability rather than *a priori* hypothesis.

**Conclusions:**

To further progress neighbourhood effects research, we suggest that more data linkage to longitudinal datasets is required beyond the narrow list identified in this review. The limited literature published to date suggests an accumulation of exposure to neighbourhood deprivation over the life course is damaging to later life health, which indicates improving neighbourhoods as early in life as possible would have the greatest public health improvement.

## Introduction

The idea that where you live can influence your health and well-being over and above your individual or household circumstances has been one of the most widely tested hypotheses in the field of health geography since the early 2000s.^1^ van Ham and Manley^2^ have suggested that the research area is at a crossroads, yet it would appear neighbourhood effects research has stalled at a roundabout given the plentiful challenges to the field that require careful navigation. van Ham and Manley^2^ suggest at least five methodological challenges, including a plea to researchers to take into account people’s neighbourhood histories. This paper reviews the current literature on life course exposure to neighbourhood deprivation and its effect on health and well-being later in life.

Another major methodological hurdle to the study of neighbourhood effects is overcoming selection bias (i.e. the selective sorting of people into neighbourhoods through choice or lack of choice). Progress on overcoming this hurdle has been slow, and the contention that neighbourhood selection is the underlying phenomenon that explains a residual neighbourhood effect remains largely unresolved.^3^ Longitudinal data have enabled researchers to overcome this to some extent. However, it is unclear whether there is a consensus on how important neighbourhoods are over the life course and if they impact more at particular time points.^4^

The lack of progress in this research area matters because governments continue to fund and facilitate area and place-based interventions and individuals spend considerable resources ensuring they live in a place that is going to benefit them most.^5–7^ Gibbons and Machin^8^ suggest individuals are willing to pay a premium over and above dwelling attributes for higher quality neighbourhood amenities. The clearest example of this process is the effect of school quality on house prices in a number of different contexts.^9–11^ Accepting the premise that where you live has no bearing on whom you become, these resources could be better spent on public interventions and individual preferences, as often only a minority of poor people live in the most deprived neighbourhoods, for example, in contexts such as the USA.^12^

A striking limitation of much of the neighbourhood effects literature is the lack of explanation of how causal mechanisms operate.^13^ Researchers are often comfortable with a single measure that captures the essence of how a neighbourhood affects their outcome of interest.^13–16^ However, failure to theorise clear causal pathways is perhaps one reason why researchers are not sure whether neighbourhood effects exist and whether selection modifies neighbourhood effects, or explains them. Galster’s^13^ work details systematically how neighbourhoods may affect individuals, with 15 causal pathways between neighbourhood and individual behavioural and health outcomes, categorized into four themes: social interactive; environmental; geographical; and institutional. But few have taken on the challenge of opening the ‘black box’ of neighbourhood effects, and Galster’s themes and pathways remain underexplored in life course data. Vocal critics of the field plead for more research that emphasizes what it is about neighbourhood that affects people living within it.^1^^,^^14^ Friedrichs *et al.*^17^ suggests that only when researchers develop specific hypotheses about mechanisms, can they arrive at adequate operationalization to test them. Prior *et al.*^18^ is a notable exception, showing the mediating effect of a stress pathway on the neighbourhood deprivation and physical health relationship.

A closely related criticism is the spatial scale of neighbourhood exposure. People’s interactions with the places they live, and work, are hard, if not impossible, to delineate. Much of the time arbitrary spatial boundaries are used to define a neighbourhood.^19^ This is important because how an area is chosen, the so-called Modifiable Areal Unit Problem (MAUP), can lead to variations in results^20^^,^^21^: the choice of spatial units determining neighbourhoods can create very different compositional and contextual characteristics. Alternative specifications of neighbourhoods increasingly appearing in the literature in Europe and the USA are more bespoke definitions created from data centred on the individual, for example, using the nearest fixed number of people, or those within a set distance.^22^ Kwan^23^^,^^24^ takes this further, suggesting individuals can experience contextual effects differently, and therefore personalized, subjective definitions of space are more appropriate than objectively defined delineations.

This review is not squarely concerned with determining the appropriate causal pathway or the spatial scale of analysis, but what the onset of rich longitudinal data geocoded to historic neighbourhood deprivation measures has done to improve the study of epidemiological neighbourhood effects research. This is the area of neighbourhood effects research where progress has been made that addresses these concerns. A fundamental limitation of many studies to date, cross-sectional in nature, is their inability to overcome the condition of temporality, i.e. the neighbourhood effect has to occur before the health outcome. Moreover, that longer exposure will be more effective than shorter exposure, a further condition missed by the point-in-time measurement in much of the neighbourhood effects literature.^25^ The problem with identifying an appropriate causal pathway and the appropriate spatial scale of effect is often data availability.^4^^,^^26^ Data are rarely rich enough to scratch beyond the surface that is required to address these concerns. The onset of longitudinal datasets has provided fruitful progress in measuring neighbourhood effects between and within generations and at critical time points during the life course to overcome problems of selection. This paper reviews this portion of the neighbourhood effects literature that is moving forward and is credited with making progress to determining appropriate causal pathways and scale effects, and where there remains much mileage in further work. Largely outside the bounds of this review because of our inclusion criteria, is the value of, for example, pseudo-experiments and natural experiments in neighbourhood effects research that have the potential to make greater strides in dealing with the problem of selection.

## Methods

### Search strategy

We searched articles published between 1 January 2010 and 28 May 2019 using Scopus and Web of Science. The period was chosen on the basis that there were almost no studies prior to 2010 with a longitudinal design that met the inclusion criteria in a preliminary search. The following search terms, or equivalents, were used: neighbourhood, effects, longitudinal and health (see Supplementary appendix for detailed search strategies). We did not specify any particular health or well-being outcome. We describe the most common outcome variables, data source used, study design, neighbourhood definitions, aggregate deprivation instrument, model covariates, modelling approach and missing data strategy across the included studies.

### Inclusion/exclusion criteria

We limited our review to those with a study period of at least 15 years between first exposure of neighbourhood and final measurement of an outcome during adulthood. This ensured we removed studies that had exclusively measured neighbourhood effects during childhood or studies that examined a relatively short-term impact of neighbourhood deprivation on health and well-being. The neighbourhood measure had to be a measure of deprivation, incorporating what some authors describe as neighbourhood poverty, socioeconomic status, disadvantage and affluence to preclude studies that exclusively focus on environmental neighbourhood hazards, for example. The environmental hazard literature is large and less spatially bound by what is commonly referred as neighbourhood. This is because pollution, for example, exposes people over a continuous space rather than fixed boundary systems typically used to represent neighbourhoods. We limited the review to English language articles but we did not specify country of study. Two reviewers identified the literature (S.J. and O.N.) and one reviewer conducted the study selection and data extraction (S.J.).

### Data extraction

The first author, year of publication, title of article and journal were used to index the studies. We also extracted the outcome, data source, study design, neighbourhood definition, neighbourhood measurement, individual co-factors, statistical model and missing data strategy. The outcome enabled us to demonstrate how the specific health measurement used in the selected studies differs from neighbourhood effects on health and well-being research more broadly. The study design enabled us to determine how the outcome and neighbourhood exposure were measured (i.e. point or trajectory). The data source timeframe enabled us to determine the period of neighbourhood effects and context. The neighbourhood definition was important to explain inconsistencies in findings due to size of spatial scale. The neighbourhood deprivation measurement was used to explain differences due to the nature of the exposure. We identified individual co-factors to indicate ability to identify neighbourhood selection confounders and potential over-adjustment. The statistical model used indicated the ability to draw causal interpretation from findings. The missing data strategy indicated the potential for attrition bias that often leads to an underestimation in effects related to socioeconomic status.^27^ A meta-analysis was not appropriate given the diversity of outcomes and methods used in the extracted studies.

**Figure 1:**
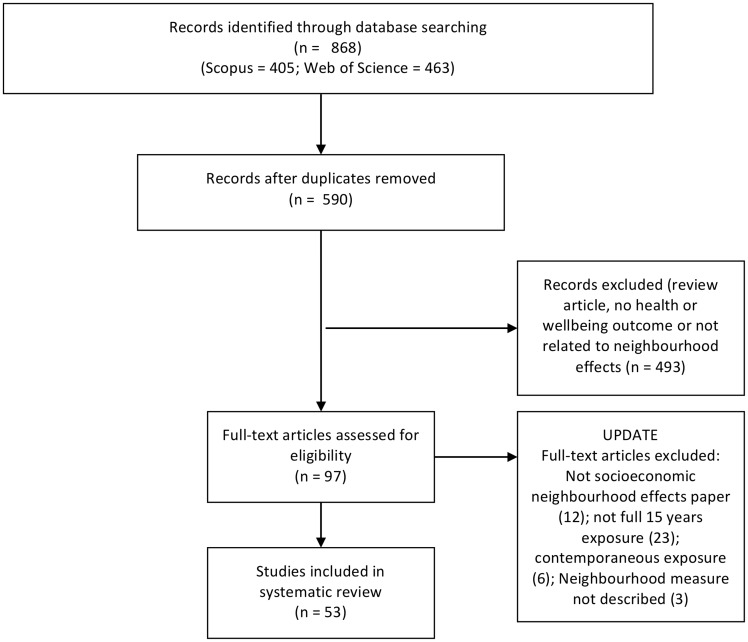
Flowchart for study selection

The studies were entered into an Excel file and descriptive statistics were produced using pivot tables (figure 1). 

## Results

The number of articles retrieved using the search terms was 868 and 53 were considered to meet the inclusion criteria. Almost half of the papers included the same researcher at least twice and 43% were published in the same three journals: *Health and Place* (10), *Social Science and Medicine* (8), *PLOS One* (5).

### Main outcome variable

The most common outcome variable, when counting more than one from studies with multiple outcomes, was mortality (18%), followed by weight gain, obesity or body mass index (BMI) (16%), health-related behaviours (15%—including smoking, alcohol and food consumption) and mental health (10%—including depression, cognition, psychosis and suicide) (see table 1). The majority of studies (74%) measured their outcome at a single point in time, whereas the others predicted trajectories (change) in their outcome. Two of the latter studies find a baseline association of neighbourhood deprivation with BMI, but little or no change over time.^28^^,^^29^ Others find declining physical health by baseline neighbourhood deprivation and cumulative exposure to neighbourhood deprivation.^30^^,^^31^

**Table 1:** Ranking of health outcomes used in reviewed studies

Health outcome	Number of studies	Percentage of total
Mortality	11	18
BMI or weight gain	10	16
Health-related behaviours	9	15
Mental health	6	10
Chronic conditions	5	8
Self-rated health	5	8
Functional somatic symptoms	4	7
Physical function	4	7
Neighbourhood disadvantage	2	3
Allostatic load	1	2
Physical activity	1	2
Preterm birth	1	2
Teenage parenthood	1	2
Grand total	61^a^	

a Includes six studies with multiple outcomes.

**Table 2:** Descriptives of neighbourhood definitions used in reviewed studies

Neighbourhood definition	Number of studies	Percentage of total	Population mean	Population range
US Census tract	23	43	4000	1200–8000
Swedish Small Area Market Statistics	10	19	1000	50–3000
US Census block	4	8	2000	600–2000
Japanese Chocho-aza	2	4	500	NA
Finnish municipality	2	4	6000	NA
Swedish municipality	2	4	30 000	NA
US counties	1	2	100 000	NA
Finnish 250 m^2^ grids	1	2	NA	10 or more
UK districts post-1974	1	2	111 000	NA
UK districts pre-1974	1	2	35 000	NA
UK middle super output areas	1	2	7000	5000–15 000
UK enumeration district	1	2	500	NA
New Zealand census area	1	2	2000	100–5000
Norwegian neighbourhood	1	2	NA	NA
Eindhoven statistical neighbourhoods	1	2	2000	NA
Perceived neighbourhood	1	2	NA	NA

NA, Not applicable.

### Data source and study design

The most common data source used was prospective survey data from the US Panel Study of Income Dynamics (PSID) (21%). More than half of the studies used data from the USA (53%). These included other prospective sample surveys: the Coronary Artery Risk Development in Young Adults (CARDIA) study (6%), the American Changing Lives (ACL) survey (6%) and the National Longitudinal Survey of Youth 1979 (NLSY79) (6%). The remaining US studies were retrospective cohort studies (3), repeated cross-sections (2) and a cross-sectional study linking current neighbourhood of residence back to the 1970 Census. The European studies were 72% panel or cohort surveys and 28% register datasets, limited to Scandinavia, the UK and the Netherlands. The most commonly used European samples were the Northern Swedish Cohort (13%) and the Young Finns Study (6%). Swedish register data were used by 10% of all studies selected including one with an experimental design.^32^ There were two studies from Japan, and one each from New Zealand and Canada.

The longest study period between neighbourhood socioeconomic exposure and outcome was measured in a British birth cohort study, the National Survey of Health and Development, when respondents were aged 4 in 1953 and then again at ages 26 and 53.^33^ The PSID also provides the possibility of a longer-term follow-up of neighbourhood exposures than most, linked from 1968 onwards.^34–38^

### Definition of neighbourhood

US Census tracts (43%) was by far the most common definition of neighbourhood, which have a mean population size of approximately 4000 people.^28^^,^^39^ A few US-based studies (8%) used census blocks containing, on average 2000 people.^31^ Many of the Swedish studies used Small-Area Market Statistics areas,^40^ which have a mean population size of 1000 (19%). The two Japanese studies used Chocho-azas, which have a median size of 400 people.^41^^,^^42^ Two UK studies used time-specific definitions of local authority districts, which have a median as large as 110 000.^33^^,^^43^ Two studies used Finish municipalities with a mean population size of 6000.^36^^,^^44^

Almost all of the studies measured the neighbourhood of residence prospectively (94%) as opposed to retrospectively (6%). The latter studies used residential life history information linked to historic census measures.^45–47^ A minority (30%) of studies explicitly made reference to using a set of spatial boundaries consistent through time derived by reapplying or reapportioning neighbourhood data from earlier and later time points.^46^^,^^48–54^

### Measurement of neighbourhood deprivation

The most common operationalization of neighbourhood deprivation was a composite measure containing multiple items, usually from a national population census (40%). These composites were mostly created by summing or taking the mean of standardized scores across indicators.^28^^,^^44^^,^^45^^,^^48^^,^^55^ The most common items included in the composites were aggregates of income, labour market participation, occupational status, welfare support and educational attainment.

Factor scores were used by a further 21% of studies derived using principal component analysis.^30^^,^^37^^,^^46^^,^^56^^,^^57^ The items used to produce factors scores were similar to those used in the composite indexes, including poverty rate/income, educational attainment, labour market participation and welfare receipt. There were five studies that used the proportion of female-headed households in their factor analysis.^30^^,^^38^^,^^46^^,^^51^^,^^57^

An alternative approach to the measurement of neighbourhood deprivation in some US-based studies was a poverty rate^58^ or poverty threshold^34^^,^^53^ derived from census data on income (15%). Other studies used single item proxies of neighbourhood poverty,^33^^,^^43^^,^^59^ neighbourhood audits^31^ and perceived neighbourhood quality.^60^

The majority of studies (64%) measured neighbourhood deprivation exposure at multiple time points rather than at one point in time earlier in the life course. The studies measuring neighbourhood deprivation once earlier in life tend to find there is an association between neighbourhood deprivation and health and well-being later in life.^34^^,^^35^^,^^58^^,^^60^^,^^61^ The time-varying exposure analyses suggest contemporaneous neighbourhood deprivation is more strongly associated with later life health and well-being but that it operates through earlier life neighbourhood deprivation^33^^,^^50^^,^^62–64^ in what is described as a chain of risk model.^65^^,^^66^ Those measuring cumulative exposure to neighbourhood deprivation find it predicts the onset and deterioration of poor health^30^^,^^40^^,^^55^^,^^67^^,^^68^ and is stronger than a contemporaneous neighbourhood effect.^25^^,^^69^ A small selection of papers explicitly test for sensitive periods when neighbourhood effects are stronger across the life course and find they are stronger at the oldest age of measurement.^62^^,^^70^^,^^71^ These studies all lend support for the chain of risk model suggesting neighbourhood deprivation exposure at one point in time is highly predictive of the subsequent measurement occasions.

### Co-varying factors

The most common factor controlled for that may explain selection into certain types of neighbourhoods was educational attainment, either the individual respondent’s or their parents’, or both (61%). Individual income, labour market participation and occupational status were other commonly used variables as potential confounders of the neighbourhood-health relationship (42%). Most of the studies controlled for age, sex or both in their analysis (58%).

There were a number of studies that controlled for prior, baseline and time-varying demographic, socioeconomic and health characteristics using multiple indicators of each in order to avoid residual confounding.^45^^,^^55^ These studies are at risk of over-adjustment (i.e. controlling for an intermediate effect on the causal pathway between exposure and outcome). Only a minority of studies formally tested for the mediating effect of variables considered confounders of the neighbourhood deprivation and health relationship.

### Modelling technique

The nature of repeated measures (i.e. longitudinal) data lends itself to multilevel modelling. Multilevel modelling can take into account the dependence of observations within a person over time and is often referred to as growth curve modelling.^72^ Two-fifths of the studies used this approach in their main analysis or in sensitivity analyses. A further 21% of studies applied modelling techniques that aim to determine causality in the relationship between time-varying covariates and a health or well-being outcome. For example, 11% of studies used fixed effects models^25^^,^^38^^,^^51^^,^^56,^^73,^^74^ and a further 9% used marginal structure models.^51^^,^^53^^,^^57^^,^^58^^,^^68^ More than a fifth of the studies used single-level linear or generalized linear models that did not explicitly take account of the temporal dependency of longitudinal data. Proportional hazard models were used to determine risk of event, usually mortality, in 11% of the studies.^35^^,^^41^^,^^42^^,^^60,^^75^

### Missing data strategy

A minority of studies (25%) addressed missing data using techniques such as multiple imputation,^51^^,^^53^^,^^57^^,^^64^^,^^68–70,^^74^ full-information maximum likelihood,^29^^,^^76^ hot-deck imputation,^52^ mean imputation^46^ and random imputation.^77^ A similar proportion of studies (26%) indicated that missing data was ignorable or likely to bias findings in a certain direction after analysing missingness. The remaining studies described the level of missingness, did nothing to address missing data without justifying whether it was necessary, or did not have missing data. There was rarely a distinction made between item non-response and sample attrition in the description of the likely biases of missing data.

## Discussion

Research on neighbourhood effects that directly attempts to rise to the challenge of causality using longitudinal data is relatively fresh. This review provides a summary of the literature of life course neighbourhood deprivation effects on health and well-being since 2010. The weight of evidence suggests neighbourhood effects accumulate over the life course when exposure to a poor socioeconomic context is sustained. This is the case for outcomes of adolescent parenthood,^53^ chronic conditions,^47^^,^^57^ disability,^43^ smoking,^68^ SRH,^51^ BMI,^25^^,^^47^^,^^57^^,^^69^ functional symptoms,^40^^,^^65^ allostatic load,^55^ mortality^58^ and physical function.^30^^,^^64^ There is a suggestion that early life neighbourhood is important, but it is often attenuated and explained by neighbourhood context later in life. Gustafsson and Sebastian^50^ suggest that this is because neighbourhood in later life is rooted in neighbourhood earlier during the life course. This may explain the considerable number of studies that found a strong contemporaneous neighbourhood effect during mid-life. There does not appear to be a strong evidence that there are sensitive periods when neighbourhood effects are stronger than other periods, except for one study suggesting neighbourhood at around age 30 directly impacts on midlife health.^70^ We should not overstate neighbourhood effects because many longitudinal studies find that only a relatively small proportion in the variance in health and well-being outcomes is attributable to the neighbourhood.^36^^,^^37^^,^^59,^^77^

The limited number of authors from the extracted studies in this review is symptomatic of the embryonic stage of life course neighbourhood effects research. This is perhaps because overcoming the challenges of doing life course neighbourhood effects research is difficult.^4^^,^^26^ These challenges are perhaps discouraging a broader pool of researchers. However, on the flip side, it is encouraging that those who have taken the plunge are getting the most out of their work. A related limitation of the field is the lack of availability of different data to test hypotheses of life course socioeconomic neighbourhood effects. More than one-third of the studies included in this review used data from two studies: PSID and Northern Swedish Cohort. The message to progress the field could not be simpler: more data and better data are required. This does not necessarily mean fresh data collection, unless it makes use of retrospective neighbourhood histories, rather linkage of other panel and cohort studies to historic aggregate census and register data.^78^ New data collections should focus on the expanding literature that uses quasi-experimental designs to test whether neighbourhoods affect health and well-being.^32^ Few of these datasets have matured sufficiently to test the length of life course neighbourhood effect, which is the concern of this review.^79^

Even with new linked data, a challenge in using longitudinal data is sample attrition, which is often systematically biased and can cause havoc with causal interpretation. When adding item non-response this can often reduce sample size by more than half since baseline.^64^^,^^70^ Conventional methods to deal with missing data in longitudinal research have progressed to an extent that they could be used more frequently in studies on life course neighbourhood effects. We find only a minority use forms of imputation that build in uncertainty under a missing at random assumption.^29^^,^^51^^,^^53^^,^^57^^,^^68^ A message from this review paper is that this should become more common and more work is required to test the assumption that longitudinal sample attrition can be explained by measured characteristics of panel study members. Moreover, when these methods are used there should be more thorough description of their purposes, for example, to correct either or both item and person non-response and the extent these are apparent as well as their association with key outcome and exposure variables.

We find a wide range of health and well-being outcomes influenced by life course neighbourhood deprivation effects. It is not surprising that mortality is the most common, given the criteria for study inclusion necessitated a measure of health outcome at least 15 years after exposure to neighbourhood. This contrasts with the neighbourhood effects literature more broadly (i.e. including the vast cross-sectional literature measuring outcome and exposure contemporaneously) which ranks obesity as the most common outcome studied, with mortality ranked 8th.^15^ Our evaluation of the nature of neighbourhood effects studies is that too many researchers are not clear on the pathways with which measures of neighbourhood socioeconomic context affects individuals. Many make reference to specific causal mechanisms and then return to their broad composite of neighbourhood quality as a proxy for the specific elements of neighbourhood that they think are important determinants of health and well-being. There is a welcome tension in neighbourhood effects research leading to fracturing into more specific areas because those who take up the challenge of identifying causality are being more precise about how places affect individuals.

The measurement of neighbourhood deprivation in the papers included in this review did not appear to bias findings in one direction or another. The most convincing studies were those that used a poverty rate to determine the socioeconomic position of the neighbourhood because it more clearly specifies a causal pathway to poorer health than a composite or factor score that incorporates disparate indicators.^34^^,^^53^^,^^58^ A number of studies used the proportion of households headed by a female as an indicator of neighbourhood socioeconomic context.^30^^,^^38^^,^^46^^,^^51^^,^^57^ It is not clear how this indicator can be causally linked to individual health and well-being.

Almost all of the studies included in this review used a definition of neighbourhood that was created by government bodies to enable enumeration or dissemination of official statistics, or for administrative purposes. There has been much criticism in other neighbourhood effects review articles questioning whether these sorts of spatial boundaries are the most appropriate scales in which to measure neighbourhood context.^15^ Our contribution to the discussion is that the findings from this review are not specific to the spatial scale of neighbourhood socioeconomic measurement. Previous calls for sensitivity analysis of multiple spatial scales, where possible, would provide more robust findings of the presence (or lack of) neighbourhood effects. Other fertile ground for further research is on the call for greater use of neighbourhoods that are based on individuals' perception of how they experience them.^23^^,^^24^^,^^60^

A specific concern highlighted by this review was the temporal mismatch between when individual and neighbourhood data were collected. Many of the studies in this review linearly interpolated census measurements to provide a neighbourhood measurement. For example, an individual data collection in 1985 when the actual neighbourhood measurement was taken at 1980 or 1990. This is problematic because it assumes no volatility in the trajectory of neighbourhoods socioeconomic context.^80^ Future validation studies could use register datasets available in countries such as Sweden to test the extent of non-linear change in neighbourhood socioeconomic context.

A clear dividing line between studies included in this review was the approach to adjustment for confounding variables of the relationship between neighbourhood socioeconomic context and health and well-being. A number of studies were guilty of a ‘kitchen-sink’ approach to their regression modelling (i.e. controlling for almost every possible variable in their available data). Over-adjustment is most likely to lead to an underestimation of neighbourhood effects because intermediate effects that lie on the causal pathway will attenuate the neighbourhood effects. In the absence of formal mediation modelling, the studies that control for a limited number of variables and concede on their ability to identify causality conclusively are more credible in our opinion. Moreover, studies should be clearer on their justification for the inclusion of confounding variables in terms of whether they reflect aspects of the very neighbourhood effects under investigation.

Additionally, there is reliance on statistical analytical methods that are appropriate for modelling trajectories in health and well-being over baseline and time-varying neighbourhood context (e.g. multilevel growth curve modelling). However, these methods do not implicitly enable researchers to claim causality. Methods that attempt to block indirect pathways to health and well-being, including those common to repeat measures analysis (e.g. fixed effects) and those coming on-stream (e.g. marginal structure modelling), were rare in this review. The infancy of life course neighbourhood effects can be demonstrated by the fact that authors using causal methods almost always provide sensitivity analysis using non-causal methods.

For researchers setting out on neighbourhood effects research, our review highlights a number of directions they can take to attempt to progress the field. Negotiating the neighbourhood effects research ‘roundabout’ is tricky because some of the exits are more clearly signposted than others. In this review article, we have detailed the attempt by a small but growing literature in the field of health geography that uses longitudinal data to determine a life course component to the neighbourhood deprivation effect thesis. One of our favoured avenues neighbourhood effects researchers could take would be to enhance existing longitudinal data sets, such as birth cohort studies, with a wider range of neighbourhood level data. This will allow a more theory driven approach to the study rather than the present largely data led approach to neighbourhood operationalization. It could be enabled by providing geocoded variables with slightly lower restriction than is commonly applied to British birth cohort study data, for example, which would allow easier linkage to neighbourhood deprivation constructs that are widely used.^70^ Some of the most pressing substantive issues that remain distinctly uncertain are whether neighbourhood deprivation in childhood causes later life poor health and well-being and whether there are sensitive periods during the life course when neighbourhood deprivation is most important. These should be addressed through analysis that determines the importance of selection into neighbourhoods across the life course.

## Funding

This work was funded by the Leverhulme Trust (RPG-2015-317) and the UCL Q-Step Centre funded by the Nuffield Foundation, the Economic and Social Research Council and the Higher Education Funding Council for England.

*Conflicts of interest*: None declared.

**Key points**

- This is the first review to bring together research on neighbourhood effects on health and well-being that takes a life course perspective.
- We find neighbourhood deprivation effects accumulate and are not particular to certain points of the life course on later life health and well-being.
- Neighbourhood effects research is critical for public health since local and national governments spend considerable amounts of resource on area and place-based interventions.
- We suggest more data linkage is required to existing longitudinal studies to expand current knowledge beyond what is known from a limited pool of research.

## Supplementary Material

ckz153_Supplementary_Data
