# Supplementary material for: The impact of life course exposures to neighbourhood deprivation on health and well-being: a review of the long-term neighbourhood effects literature
Source: Eur J Public Health. 2019 Oct 2;30(5):922–8. doi: 10.1093/eurpub/ckz153 (PMC8489013; doi:10.1093/eurpub/ckz153)
Supplement: ckz153_Supplementary_Data [file ckz153_supplementary_data.zip › ckz153-suppl_data/ejph-2018-12-srm-1092-File003.docx]

Additional references

41. 41. Miki Y, Inoue M, Ikeda A, Sawada N, Nakaya T, Shimazu T. Neighborhood Deprivation and Risk of Cancer Incidence , Mortality and Survival : Results from a Population-Based Cohort Study in Japan. 2014;9(9). doi:10.1371/journal.pone.0106729

42. Nakaya T, Honjo K, Hanibuchi T, Ikeda A, Iso H, Inoue M. Associations of All-Cause Mortality with Census-Based Neighbourhood Deprivation and Population Density in Japan : A Multilevel Survival Analysis. 2014;9(6). doi:10.1371/journal.pone.0097802

43. Riva M, Curtis SE. Long-term local area employment rates as predictors of individual mortality and morbidity: a prospective study in England, spanning more than two decades. *J Epidemiol Community Health*. January 2012.

44. Airaksinen J, Hakulinen C, Elovainio M, et al. Neighborhood effects in depressive symptoms, social support, and mistrust: Longitudinal analysis with repeated measurements. *Soc Sci Med*. 2015;136-137:10-16. doi:https://doi.org/10.1016/j.socscimed.2015.04.034

45. Hystad P, Carpiano RM, Demers PA, Johnson KC, Brauer M. Neighbourhood socioeconomic status and individual lung cancer risk: Evaluating long-term exposure measures and mediating mechanisms. *Soc Sci Med*. 2013;97(SI):95-103. doi:10.1016/j.socscimed.2013.08.005

46. Osypuk TL, Kehm R, Misra DP. Where We Used to Live: Validating Retrospective Measures of Childhood Neighborhood Context for Life Course Epidemiologic Studies. Chang C-K, ed. *PLoS One*. 2015;10(4):e0124635. doi:10.1371/journal.pone.0124635

47. Murray ET, Roux AVD, Carnethon M, Lutsey PL, Ni H, O’Meara ES. Trajectories of Neighborhood Poverty and Associations With Subclinical Atherosclerosis and Associated Risk Factors. *Am J Epidemiol*. 2010;171(10):1099-1108. doi:10.1093/aje/kwq044

48. Crowder K, South SJ. Spatial and temporal dimensions of neighborhood effects on high school graduation. *Soc Sci Res*. 2011;40(1):87-106. doi:10.1016/j.ssresearch.2010.04.013

49. Naess O, Leyland AH. Analysing the effect of area of residence over the life course in multilevel epidemiology. *Scand J Public Health*. 2010;38(5 Suppl):119-126. doi:10.1177/1403494810384646

50. Pearson AL, Apparicio P, Riva M. Cumulative disadvantage ? Exploring relationships between neighbourhood deprivation trends ( 1991 to 2006 ) and mortality in New Zealand Cumulative disadvantage ? Exploring relationships between neighbourhood deprivation trends ( 1991 to 2006 ) and mortality in New Zealand. 2013.

51. Kravitz-Wirtz N. Cumulative Effects of Growing Up in Separate and Unequal Neighborhoods on Racial Disparities in Self-rated Health in Early Adulthood. *J Health Soc Behav*. 2016;57(4):453-470. doi:10.1177/0022146516671568

52. Walsemann KM, Child S, Heck K, et al. Are the poverty histories of neighbourhoods associated with psychosocial well-being among a representative sample of California mothers? An observational study. *J Epidemiol Community Health*. 2017;71(6):558 LP - 564.

53. Wodtke GT. Duration and Timing of Exposure to Neighborhood Poverty and the Risk of Adolescent Parenthood. *Demography*. 2013;50(5):1765-1788. doi:10.1007/s13524-013-0219-z

54. Margerison-Zilko C, Cubbin C, Jun J, Marchi K, Fingar K, Braveman P. Beyond the Cross-Sectional: Neighborhood Poverty Histories and Preterm Birth. *Am J Public Health*. 2015;105(6):1174-1180. doi:10.2105/AJPH.2014.302441

55. Gustafsson PE, San Sebastian M, Janlert U, Theorell T, Westerlund H, Hammarström A. Life-course accumulation of neighborhood disadvantage and allostatic load: empirical integration of three social determinants of health frameworks. *Am J Public Health*. 2014;104(5):904-910. doi:10.2105/AJPH.2013.301707

56. Boone-Heinonen J, Roux AVD, Kiefe CI, Lewis CE, Guilkey DK, Gordon-Larsen P. Neighborhood socioeconomic status predictors of physical activity through young to middle adulthood: The CARDIA study. *Soc Sci Med*. 2011;72(5):641-649. doi:10.1016/j.socscimed.2010.12.013

57. Kravitz-Wirtz N. Temporal Effects of Child and Adolescent Exposure to Neighborhood Disadvantage on Black/White Disparities in Young Adult Obesity. *J Adolesc Heal*. 2016;58(5):551-557. doi:10.1016/j.jadohealth.2016.01.004

58. Do DP, Wang L, Elliott MR. Investigating the relationship between neighborhood poverty and mortality risk: A marginal structural modeling approach. *Soc Sci Med*. 2013;91(SI):58-66. doi:10.1016/j.socscimed.2013.03.003

59. Dundas R, Leyland AH, Macintyre S. Early-life school, neighborhood, and family influences on adult health: a multilevel cross-classified analysis of the Aberdeen children of the 1950s study. *Am J Epidemiol*. 2014;180(2):197-207. doi:10.1093/aje/kwu110

60. Assari S. Perceived Neighborhood Safety Better Predicts Risk of Mortality for Whites than Blacks. *J Racial Ethn Heal Disparities*. 2017:937-948. doi:10.1007/s40615-016-0297-x

61. Gustafsson PE, San Sebastian M, Janlert U, Theorell T, Westerlund H, Hammarström A. Residential selection across the life course: adolescent contextual and individual determinants of neighborhood disadvantage in mid-adulthood. *PLoS One*. 2013;8(11):e80241. doi:10.1371/journal.pone.0080241

62. Gustafsson PE, Bozorgmehr K, Hammarstrom A, San Sebastian M. What role does adolescent neighborhood play for adult health? A cross-classified multilevel analysis of life course models in Northern Sweden. *Health Place*. 2017;46:137-144. doi:10.1016/j.healthplace.2017.04.013

63. Gustafsson PE, San Sebastian M. When does hardship matter for health? Neighborhood and individual disadvantages and functional somatic symptoms from adolescence to mid-life in the Northern Swedish Cohort. *PLoS One*. 2014;9(6). doi:10.1371/journal.pone.0099558

64. Kail BL, Spring A, Gayman M. A Conceptual Matrix of the Temporal and Spatial Dimensions of Socioeconomic Status and Their Relationship with Health. *Journals Gerontol - Ser B Psychol Sci Soc Sci*. 2019;74(1):148-159. doi:10.1093/geronb/gby025

65. Jonsson F, Sebastian MS, Hammarström A, Gustafsson PE. Are neighbourhood inequalities in adult health explained by socio-economic and psychosocial determinants in adolescence and the subsequent life course in northern Sweden? A decomposition analysis. *Heal Place*. 2018;52:127-134. doi:10.1016/j.healthplace.2018.05.010

66. Karriker-Jaffe KJ, Lönn SL, Cook WK, Kendler KS, Sundquist K. Chains of risk for alcohol use disorder: Mediators of exposure to neighborhood deprivation in early and middle childhood. *Heal Place*. 2018;50:16-26. doi:10.1016/j.healthplace.2017.12.008

67. White JS, Hamad R, Li X, et al. Long-term effects of neighbourhood deprivation on diabetes risk: quasi-experimental evidence from a refugee dispersal policy in Sweden. *Lancet Diabetes Endocrinol*. 2016;4(6):517-524. doi:https://doi.org/10.1016/S2213-8587(16)30009-2

68. Kravitz-Wirtz N. A discrete-time analysis of the effects of more prolonged exposure to neighborhood poverty on the risk of smoking initiation by age 25. *Soc Sci Med*. 2016;148:79-92. doi:10.1016/j.socscimed.2015.11.027

69. Headen I, Mujahid M, Deardorff J, Rehkopf DH, Abrams B. Associations between cumulative neighborhood deprivation, long-term mobility trajectories, and gestational weight gain. *Heal Place*. 2018;52:101-109. doi:10.1016/j.healthplace.2018.05.007

70. Jivraj S, Nicholas O, Murray ET, Norman P. Are there sensitive neighbourhood effect periods during the life course on midlife health and wellbeing? *Heal Place*. 2019.

71. Murray ET, Ben-Shlomo Y, Tilling K, et al. Area Deprivation Across the Life Course and Physical Capability in Midlife: Findings From the 1946 British Birth Cohort. *Am J Epidemiol*. 2013;178(3):441-450. doi:10.1093/aje/kwt003

72. Langford IH, Leyland AH, Rasbash J, Goldstein H. Multilevel Modelling of the Geographical Distributions of Diseases. *J R Stat Soc Ser C (Applied Stat*. 1999;48(2):253. http://search.ebscohost.com/login.aspx?direct=true&db=bth&AN=4519887&site=ehost-live.

73. Rummo PE, Meyer KA, Green A, Shikany JM, Guilkey DK, Gordon-larsen P. Health & Place Fast food price , diet behavior , and cardiometabolic health : Differential associations by neighborhood SES and neighborhood fast food restaurant availability in the CARDIA study. *Health Place*. 2015;35:128-135. doi:10.1016/j.healthplace.2015.06.010

74. Hamad R, Brown DM, Basu S. The association of county-level socioeconomic factors with individual tobacco and alcohol use: a longitudinal study of US adults. *BMC Public Health*. 2019;19. doi:10.1186/s12889-019-6700-x

75. Beenackers MA, Oude Groeniger J, Kamphuis CBM, Van Lenthe FJ. Urban population density and mortality in a compact Dutch city: 23-year follow-up of the Dutch GLOBE study. *Heal Place*. 2018;53:79-85. doi:10.1016/j.healthplace.2018.06.010

76. Lee JO, Jones TM, Kosterman R, et al. Childhood neighborhood context and adult substance use problems: the role of socio-economic status at the age of 30 years. *Public Health*. 2018;165:58-66. doi:10.1016/j.puhe.2018.09.011

77. Zammit S, Lewis G, Rasbash J, Dalman C, Gustafsson J, Allebeck P. Individuals, schools, and neighborhood: A multilevel longitudinal study of variation in incidence of psychotic disorders. *Arch Gen Psychiatry*. 2010;67(9):914-922.

78. Schinasi LH, Auchincloss AH, Forrest CB, Diez Roux A V. Using electronic health record data for environmental and place based population health research: a systematic review. *Ann Epidemiol*. 2018;28(7):493-502. doi:https://doi.org/10.1016/j.annepidem.2018.03.008

79. Rudolph KE, Sofrygin O, Schmidt NM, et al. Mediation of neighborhood effects on adolescent substance use by the school and peer environments. *Epidemiology*. 2018;29(4):590-598. doi:10.1097/EDE.0000000000000832

80. Nicholas O. How Valid are Decennial Census Measures of Neighbourhoods in Assessing Neighbourhood Effects? In: *Association of American Geographers Annual Meeting, 5-9 April*. Boston, USA: Association of American Geographers; 2017.
