# Supplementary material for: The impact of life course exposures to neighbourhood deprivation on health and well-being: a review of the long-term neighbourhood effects literature
Source: Eur J Public Health. 2019 Oct 2;30(5):922–8. doi: 10.1093/eurpub/ckz153 (PMC8489013; doi:10.1093/eurpub/ckz153)
Supplement: ckz153_Supplementary_Data [file ckz153_supplementary_data.zip › ckz153-suppl_data/ejph-2018-12-srm-1092-File004.docx]

**Appendix**

**Scopus search terms**

( TITLE-ABS-KEY ( "neighbourhood effects"  OR  "neighbourhood composition"  OR  "neighbourhood deprivation"  OR  "neighbourhood poverty"  OR  "neighbourhood context"  OR  "neighbourhood disadvantage"  OR  "neighbourhood affluence"  OR  "neighbourhood socioeconomic" )  AND  TITLE-ABS-KEY ( longitudinal  OR  "life course"  OR  "lifecourse"  OR  "life-course"  OR  "over time"  OR  temporal )  AND  TITLE-ABS-KEY ( health  OR  wellbeing ) )  AND  ( LIMIT-TO ( PUBYEAR ,  2018 )  OR  LIMIT-TO ( PUBYEAR ,  2017 )  OR  LIMIT-TO ( PUBYEAR ,  2016 )  OR  LIMIT-TO ( PUBYEAR ,  2015 )  OR  LIMIT-TO ( PUBYEAR ,  2014 )  OR  LIMIT-TO ( PUBYEAR ,  2013 )  OR  LIMIT-TO ( PUBYEAR ,  2012 )  OR  LIMIT-TO ( PUBYEAR ,  2011 )  OR  LIMIT-TO ( PUBYEAR ,  2010 ) )

**Web of knowledge search terms**

TS= ("neighborhood effects" OR "neighborhood composition" OR "neighborhood deprivation" OR "neighborhood poverty" OR "neighborhood context" Or "neighborhood disadvantage" OR "neighborhood affluence" OR "neighborhood socioeconomic”) AND TS=(longitudinal OR "life course" OR "lifecourse" OR "life-course" OR "over time" OR temporal) AND TS=(health OR wellbeing)

TS= ("neighbourhood effects" OR "neighbourhood composition" OR "neighbourhood deprivation" OR "neighbourhood poverty" OR "neighbourhood context" Or "neighbourhood disadvantage" OR "neighbourhood affluence" OR "neighbourhood socioeconomic”) AND TS=(longitudinal OR "life course" OR "lifecourse" OR "life-course" OR "over time" OR temporal) AND TS=(health OR wellbeing)

Selected study characteristics

| First author | Year | Title | Journal | Country | Design I | Sample | Point or trajectory | Outcome | NH definition | NH measure | Model I | | Missing data | | |
| --- | --- | --- | --- | --- | --- | --- | --- | --- | --- | --- | --- | --- | --- | --- | --- |
| Airaksinen | 2015 | Neighborhood effects in depressive symptoms, social support, and mistrust: Longitudinal analysis with repeated measurements | Social Science & Medicine | Finland | prospective cohort | Young Finns | outcome trajectory, exposure trajectory | Mental health | Finish Municipality | Composite score | RE | | 0 | | |
| Airaksinen | 2016 | Neighbourhood effects in health behaviours: a test of social causation with repeat-measurement longitudinal data | European Journal of Public Health | Finland | prospective cohort | Young Finns | outcome trajectory, exposure trajectory | Smoking and drinking | Finish Municipality | Composite score | RE | | 2 | | |
| Assari | 2017 | Perceived Neighborhood Safety Better Predicts Risk of Mortality for Whites than Blacks | Journal of Racial and Ethnic Health Disparities | USA | prospective panel | ACL | outcome point, exposure point | Mortality | Perceived neighbourhood | Perceived neighbourhood quality | Proportional hazard | | 0 | | |
| Boone-Heinonen | 2011 | Neighborhood socioeconomic status predictors of physical activity through young to middle adulthood: The CARDIA study | Social Science & Medicine | USA | prospective panel | CARDIA | outcome trajectory, exposure trajectory | Physical activity | Census tract | Factor score | FE repeated | | 2 | | |
| Clarke | 2015 | Cognitive decline and the neighborhood environment | Annals of Epidemiology | USA | prospective panel | Chicago | outcome trajectory, exposure point | Mental health | Census block | Neighourhood audit | RE | | 0 | | |
| Clarke | 2014 | Cumulative Exposure to Neighborhood Context: Consequences for Health Transitions Over the Adult Life Course | Research on Aging | USA | prospective panel | ACL | outcome trajectory, exposure trajectory | Physical function | Census tract | Factor score | Multinomial | | 2 | | |
| Crowder | 2010 | Spatial and temporal dimensions of neighborhood effects on high school graduation q | Social Science Research | USA | prospective panel | PSID | outcome point, exposure trajectory | School graduation | Census tract |  | | 0 | | |  |
| Do | 2014 | Investigating the relationship between neighborhood poverty and mortality risk: A marginal structural modeling approach | Social Science & Medicine | USA | prospective panel | PSID | outcome point, exposure point | Mortality | Census tract | Census poverty rate | Marginal structure model | | 1 | | |
| Dundas | 2015 | Early-Life School, Neighborhood, and Family Influences on Adult Health: A Multilevel Cross-Classified Analysis of the Aberdeen Children of the 1950s Study | American Journal of Epidemiology | UK | prospective cohort | Aberdeen cohort study | outcome point, exposure point | Self-rated health | Enuermation district | Item scores | RE cross classified | | 0 | | |
| Gustafsson | 2014 | Life-Course Accumulation of Neighborhood Disadvantage and Allostatic Load: Empirical Integration of Three Social Determinants of Health Frameworks | Research and Practice | Sweden | prospective cohort | NSC | outcome point, exposure trajectory | Allostatic load | SAMS | Composite score | OLS | | 1 | | |
| Gustafsson | 2017 | What role does adolescent neighborhood play for adult health? A cross- classified multilevel analysis of life course models in Northern Sweden | Health & Place | Sweden | prospective cohort | NSC | outcome point, exposure trajectory | Functional somatic symptoms | SAMS | Composite score | RE cross classified | | 2 | | |
| Gustafsson | 2015 | Cumulative contextual and individual disadvantages over the life course and adult functional somatic symptoms in Sweden | European Journal of Public Health | Sweden | prospective cohort | NSC | outcome point, exposure trajectory | Functional somatic symptoms | SAMS | Composite score | OLS | | 2 | | |
| Gustafsson | 2015 | When Does Hardship Matter for Health? Neighborhood and Individual Disadvantages and Functional Somatic Symptoms from Adolescence to Mid-Life in the Northern Swedish Cohort | PLOS One | Sweden | prospective cohort | NSC | outcome point, exposure trajectory | Functional somatic symptoms | SAMS | Composite score | RE | | 2 | | |
| Gustafsson | 2013 | Residential Selection across the Life Course: Adolescent Contextual and Individual Determinants of Neighborhood Disadvantage in Mid-Adulthood | PLOS One | Sweden | prospective cohort | NSC | outcome point, exposure point | Neighbourhood disadvantage | SAMS | Composite score | RE | | 0 | | |
| Hystad | 2013 | Neighbourhood socioeconomic status and individual lung cancer risk: Evaluating long-term exposure measures and mediating mechanisms | Social Science & Medicine | USA | retrospective cohort study | National Enhanced Cancer Surveillance System sampling frame | outcome point, exposure trajectory | Chronic conditions | Census tract | Composite score | RE | | 0 | | |
| Johnansson | 2015 | Neighbourhood disadvantage and individual adversities in adolescence and total alcohol consumption up to mid-life—Results from the Northern Swedish Cohort | Health & Place | Sweden | prospective cohort | NSC | outcome trajectory, exposure point | Smoking and drinking | SAMS | Composite score | 2 | | |  |  |
| Johnson | 2012 | Health disparities in mid-to-late life: The role of earlier life family and neighborhood socioeconomic conditionsq | Social Science & Medicine | USA | prospective panel | PSID | outcome trajectory, exposure point | Self-rated health | Census block | Poverty thresholds | RE | | 2 | | |
| Johnson | 2011 | Early-Life Origins of Adult Disease: National Longitudinal Population-Based Study of the United States | Research and Practice | USA | prospective panel | PSID | outcome point, exposure point | Multi-outcome | Census tract | Poverty thresholds | Proportional hazard | | 2 | | |
| Kravitz-Wirtz | 2016 | Temporal Effects of Child and Adolescent Exposure to Neighborhood Disadvantage on Black/White Disparities in Young Adult Obesity | Journal of Adolescent Health | USA | prospective panel | PSID | outcome point, exposure trajectory | BMI | Census tract | Factor score | Marginal structure model | | 3 | | |
| Kravitz-Wirtz | 2017 | Cumulative Effects of Growing Up in Separate and Unequal Neighborhoods on Racial Disparities in Self-rated Health in Early Adulthood | Journal of Health and Social Behavior | USA | prospective panel | PSID | outcome point, exposure trajectory | Self-rated health | Census tract | Factor score | Marginal structure model | | 3 | | |
| Kravitz-Wirtz | 2016 | A discrete-time analysis of the effects of more prolonged exposure to neighborhood poverty on the risk of smoking initiation by age 25 | Social Science & Medicine | USA | prospective panel | PSID | outcome point, exposure trajectory | Smoking and drinking | Census tract | Poverty thresholds | Marginal structure model | | 3 | | |
| Marcus | 2016 | The joint contribution of neighborhood poverty and social integration to mortality risk in the United States | Annals of Epidemiology | USA | Repeated cross-sections | NHANES | outcome point, exposure point | Mortality | Census tract | Poverty thresholds | Proportional hazard | | 2 | | |
| Margerison-Zilko | 2015 | Beyond the Cross-Sectional: Neighborhood Poverty Histories and Preterm Birth | Research and Practice | USA | Repeated cross-sections | MIHA | outcome point, exposure trajectory | Preterm birth | Census tract |  | | 1 | | |  |
| Micheal | 2014 | Does change in the neighborhood environment prevent obesity in older women? | Social Science & Medicine | USA | prospective cohort | Portland | outcome trajectory, exposure point | BMI | Census block | Composite score | Latent growth model | | 3 | | |
| Miki | 2015 | Neighborhood Deprivation and Risk of Cancer Incidence, Mortality and Survival: Results from a Population-Based Cohort Study in Japan | PLOS One | Japan | prospective cohort | JPHC | outcome point, exposure point | Mortality | Chocho-aza | Composite score | Proportional hazard | | 0 | | |
| Murray | 2013 | Area Deprivation Across the Life Course and Physical Capability in Midlife: Findings From the 1946 British Birth Cohort | American Journal of Epidemiology | UK | prospective cohort | NSHD | outcome point, exposure trajectory | Physical function | Districts | Item score | RE cross classified | | 1 | | |
| Naess | 2010 | Analysing the effect of area of residence over the life course in multilevel epidemiology | Scandinavian Journal of Public Health | Norway | Register data | Olso Mortality Study | outcome point, exposure trajectory | Mortality | 1960 Norweigan Census areas | None | RE cross classified | | n/a | | |
| Nakaya | 2015 | Associations of All-Cause Mortality with Census-Based Neighbourhood Deprivation and Population Density in Japan: A Multilevel Survival Analysis | PLOS One | Japan | prospective | JPHC | outcome point, exposure point | Mortality | Chocho-aza | Composite score | Proportional hazard | | 0 | | |
| Osypuk | 2015 | Where We Used to Live: Validating Retrospective Measures of Childhood Neighborhood Context for Life Course Epidemiologic Studies | PLOS One | USA | retrospective cohort study | LIFE | outcome point, exposure trajectory | Neighbourhood disadvantage | Census tract | Factor score | Linear | | 3 | | |
| Pearson | 2014 | Cumulative disadvantage? Exploring relationships between neighbourhood deprivation trends (1991 to 2006) and mortality in New Zealand | International Journal of Health Geographics | New Zealand | Aggregate | NZ Census | outcome point, exposure trajectory | Mortality | New Zealand Census area | Latent class growth model | Negative binomial | | n/a | | |
| Richardson | 2014 | Neighborhood socioeconomic status and food environment: A 20-year longitudinal latent class analysis among CARDIA participants | Health & Place | USA | prospective cohort | CARDIA | outcome trajectory, exposure trajectory | Fast food | Census tract | Latent class growth model | Latent class growth model | |  | | |
| Riva | 2012 | Long-term local area employment rates as predictors of individual mortality and morbidity: a prospective study in England, spanning more than two decades | JECH | UK | prospective panel | ONSLS | outcome point, exposure trajectory | Mortality | Districts | Item score | RE | | 2 | | |
| Ruel | 2010 | Neighborhood effects on BMI trends: Examining BMI trajectories for Black and White women | Health & Place | USA | prospective panel | ACL | outcome trajectory, exposure point | BMI | Census tract |  | | 2 | | |  |
| Rummo | 2015 | Fast food price, diet behavior, and cardiometabolic health: Differential associations by neighborhood SES and neighborhood fast food restaurant availability in the CARDIA study | Health & Place | USA | prospective cohort | CARDIA | outcome trajectory, exposure trajectory | BMI | Census tract | Factor score | FE repeated | | 1 | | |
| Vartanian | 2012 | The Effects of Childhood SNAP Use and Neighborhood Conditions on Adult Body Mass Index | Demography | USA | prospective panel | PSID | outcome point, exposure point | BMI | Census tract | Factor score | 0 | | |  |  |
| Vartanian | 2010 | The Effects of Childhood Neighborhood Conditions on Self-reports of Adult Health | Journal of Health and Social Behavior | USA | prospective panel | PSID | outcome trajectory, exposure trajectory | Self-rated health | Census tract | Factor score | FE repeated | | 0 | | |
| Walsemann | 2017 | Are the poverty histories of neighbourhoods associated with psychosocial well-being among a representative sample of California mothers? An observational study | JECH | USA | Cross-sectional survey | MIHA | outcome point, exposure trajectory | Mental health | Census tract | Poverty thresholds | Logistic | | 3 | | |
| White | 2016 | Long-term effects of neighbourhood deprivation on diabetes risk: quasi-experimental evidence from a refugee dispersal policy in Sweden | Lancet Diabetes Endocrinol | Sweden | Experimental | Swedish Register data | outcome point, exposure point | Chronic conditions | SAMS | Factor score | FE repeated | | 1 | | |
| Wodtke | 2013 | Duration and Timing of Exposure to Neighborhood Poverty and the Risk of Adolescent Parenthood | Demography | USA | prospective panel | PSID | outcome point, exposure trajectory | Teenage parenthood | Census tract | Poverty thresholds | Marginal structure model | | 3 | | |
| Zammit | 2014 | Individual- and area-level influence on suicide risk: a multilevel longitudinal study of Swedish schoolchildren | Psychological Medicine | Sweden | prospective registers | Swedish Register data | outcome point, exposure point | Mental health | Swedish Municipality | Composite score | RE | | 1 | | |
| Zammit | 2010 | Individuals, Schools, and Neighborhood | Arch Gen Psychiatry | Sweden | prospective registers | Swedish Register data | outcome point, exposure point | Mental health | Swedish Municipality | Composite score | RE | | 3 | | |
| Lee | 2018 | Childhood neighborhood context and adult substance use problems: the role of socio-economic status at the age of 30 years | Public Health | USA | prospective panel | Seattle Social Development Project | outcome point, exposure point | Smoking and drinking | Census block | Factor score | Negative binomial | | 3 | | |
| Yang | 2018 | Neighborhood effects on body mass: Temporal and spatial dimensions | Social Science & Medicine | USA | prospective cohort | NLSY79 | outcome trajectory, exposure trajectory | BMI | Census tract | Census poverty rate | FE repeated | | 0 | | |
| Headen | 2018 | Associations between cumulative neighborhood deprivation, long-term T mobility trajectories, and gestational weight gain | Health & Place | USA | prospective cohort | NLSY79 | outcome point, exposure trajectory | BMI | Census tract | Factor score | Multinomial | | 3 | | |
| Karriker-Jaffe | 2018 | Chains of risk for alcohol use disorder: Mediators of exposure to neighborhood deprivation in early and middle childhood | Health & Place | Sweden | prospective registers | Swedish Register data | outcome point, exposure trajectory | Smoking and drinking | SAMS | Composite score | SEM | |  | | |
| Jonsson | 2018 | Are neighbourhood inequalities in adult health explained by socio-economic T and psychosocial determinants in adolescence and the subsequent life course in northern Sweden? A decomposition analysis | Health & Place | Sweden | prospective cohort | NSC | outcome point, exposure trajectory | Functional somatic symptoms | SAMS | Composite score | RE | | 0 | | |
| Beenackers | 2018 | Urban population density and mortality in a compact Dutch city: 23-year T follow-up of the Dutch GLOBE study | Health & Place | Netherlands | prospective cohort | GLOBE | outcome point, exposure point | Mortality | Statistical neighbourhoods | Item score | Proportional hazard | | 1 | | |
| Kivimäki | 2018 | Neighbourhood socioeconomic disadvantage, risk factors, and diabetes from childhood to middle age in the Young Finns Study: a cohort study | Lancet Public Health | Finland | prospective cohort | Young Finns | outcome point, exposure trajectory | Multi-outcome | Grid square | Composite score | RE | | 2 | | |
| Åström | 2018 | Differences in declining mortality rates due to coronary heart disease by neighbourhood deprivation | JECH | Sweden | Aggregate | Swedish Register data | outcome point, exposure trajectory | Mortality | SAMS | Composite score | Joinpoint regression | | n/a | | |
| Hamad | 2019 | The association of county-level socioeconomic factors with individual tobacco and alcohol use: a longitudinal study of U.S. adults | BMC Public Health | USA | prospective cohort | NLSY79 | outcome trajectory, exposure trajectory | Smoking and drinking | US Counties | Item scores | FE repeated | | 3 | | |
| Kail | 2019 | A Conceptual Matrix of the Temporal and Spatial Dimensions of Socioeconomic Status and Their Relationship with Health | Journals of Gerontology: Social Sciences | USA | prospective panel | PSID | outcome point, exposure trajectory | Physical function | Census tract | Item score | Negative binomial | | 3 | | |
| Jivraj | 2019 | Are there sensitive neighbourhood effect periods during the life course on midlife health and wellbeing? | Health & Place | UK | prospective cohort | NCDS/BCS70 | outcome point, exposure trajectory | Multi-outcome | MSOA | Composite score | RE cross classified | | 3 | | |
| Murray | 2010 | Trajectories of Neighborhood Poverty and Associations With Subclinical Atherosclerosis and Associated Risk Factors | American Journal of Epidemiology | USA | retrospective cohort study | MESA | outcome point, exposure trajectory | Multi-outcome | Census tract | Poverty thresholds | Latent growth model | | 2 | | |
